# Minimizing Ischemia Reperfusion Injury in Xenotransplantation

**DOI:** 10.3389/fimmu.2021.681504

**Published:** 2021-09-09

**Authors:** Parth M. Patel, Margaret R. Connolly, Taylor M. Coe, Anthony Calhoun, Franziska Pollok, James F. Markmann, Lars Burdorf, Agnes Azimzadeh, Joren C. Madsen, Richard N. Pierson

**Affiliations:** ^1^Department of Surgery, Center for Transplantation Sciences, Massachusetts General Hospital and Harvard Medical School, Boston, MA, United States; ^2^Department of Surgery, Division of Cardiac Surgery, Massachusetts General Hospital and Harvard Medical School, Boston, MA, United States; ^3^Department of Anesthesiology, University Hospital Hamburg-Eppendorf, Hamburg, Germany; ^4^Department of Surgery, Division of Transplantation, Massachusetts General Hospital and Harvard Medical School, Boston, MA, United States

**Keywords:** xenotranplantation, ischemia reperfusion (I/R) injury, *ex vivo* perfusion, initial xenograft dysfunction, ischemia reperfusion injury mechanisms, ischemia reperfusion injury minimization

## Abstract

The recent dramatic advances in preventing “initial xenograft dysfunction” in pig-to-non-human primate heart transplantation achieved by minimizing ischemia suggests that ischemia reperfusion injury (IRI) plays an important role in cardiac xenotransplantation. Here we review the molecular, cellular, and immune mechanisms that characterize IRI and associated “primary graft dysfunction” in allotransplantation and consider how they correspond with “xeno-associated” injury mechanisms. Based on this analysis, we describe potential genetic modifications as well as novel technical strategies that may minimize IRI for heart and other organ xenografts and which could facilitate safe and effective clinical xenotransplantation.

## Introduction

Xenotransplantation has historically been studied by scientists and physicians as an appealing potential solution to numerous medical ailments. The earliest documented xenotransplant is the 17^th^ century animal blood transfusion into humans (1, 2). Solid organ xenotransplantation studies began in the mid 19th century with the development of vascular anastomotic techniques and *ex vivo* machine perfusion (3). The current emphasis on using porcine xenografts in humans arose in the 1990s, and stimulated development of pig-to-non-human primate pre-clinical models (4–6). Simultaneously, genetic modification of pigs by nuclear deoxyribonucleic acid (DNA) microinjection coupled with advances in *in vitro* fertilization of large mammals raised optimism that genetic engineering of source pigs could help overcome the formidable immunological barriers identified in early solid organ xenotransplantation efforts (7). While these efforts have generated remarkable progress over the last 25 years, recent evidence points to a particularly important role for ischemia reperfusion injury in the initial graft dysfunction often observed following whole organ xenotransplantation.

The concept of “ischemia” has been known for millennia; originating from the ancient Greek term “ischaimos,” meaning to restrain blood. However, the idea that reperfusion, rather than the ischemic event itself, triggers many of the adverse consequences associated with ischemia is relatively new, enabled by Alexis Carrell’s development of vascular surgical, organ transplant, and organ perfusion techniques that allowed restoration of flow to an ischemic limb or organ (5, 6, 8–11). Effects of reperfusion after acute myocardial infarction (MI) and associated with ischemic limb, kidney, and liver revascularization were first studied at a mechanistic level in the early 1970s (12–16). Critical ischemia reperfusion injury (IRI) pathways were discovered or better understood based on work in the 1980’s, which defined key roles for integrins, selectins, and complement, as well as involvement of diverse cell death pathways (12–20). In parallel, IRI and techniques of ischemia reperfusion injury minimization (IRIM) have been studied extensively in solid organ allotransplantation (21–24). Herein, we review the literature regarding the mechanisms of IRI following allotransplantation in order to better understand how they may correspond with known mechanisms of xenograft injury. We identify applications of IRIM techniques, as well as genetic and pharmacologic approaches, that could facilitate effective clinical use of organ xenografts.

## Mechanisms of IRI

**Figure 1:**
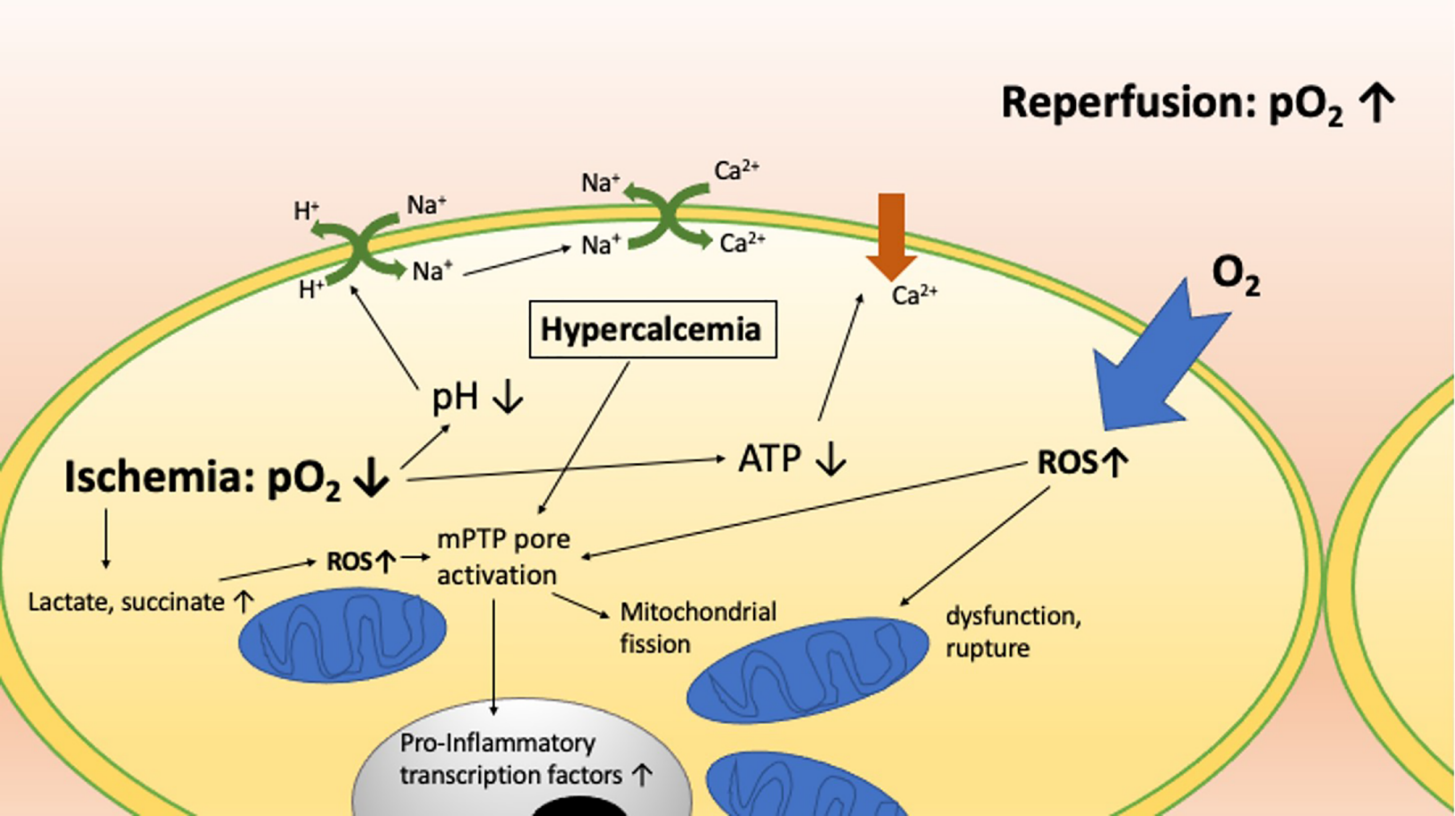
The molecular mechanisms involved in ischemia reperfusion injury and how they interact with one another.

**Figure 2:**
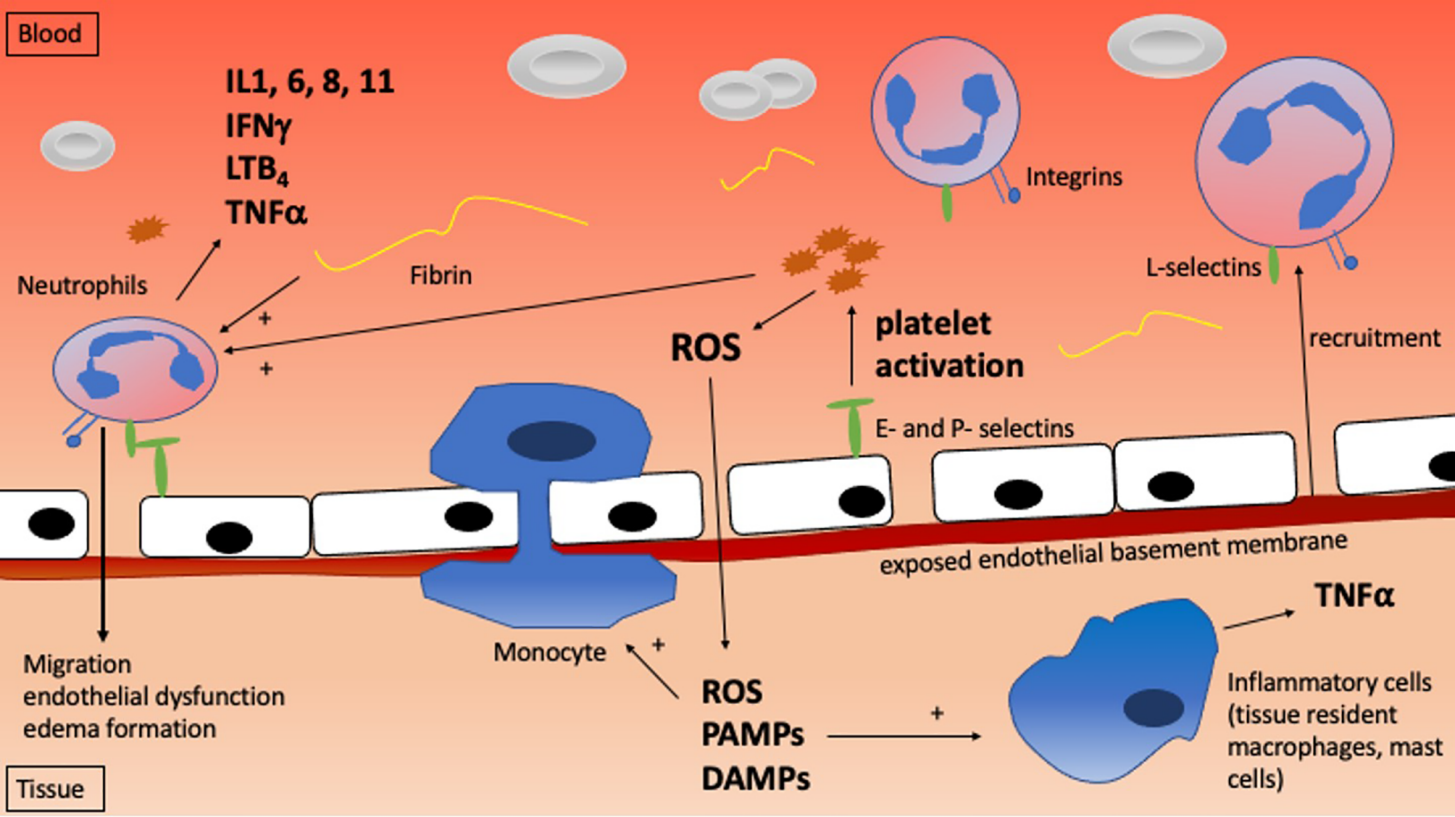
The systemic mechanisms involved with ischemia reperfusion injury.

Understanding the mechanisms that mediate IRI and IRI-driven cell death is critical to developing strategies to avoid it. IRI ranges in severity from minor and transient to severe and life-threatening, reflecting variable degrees of cell and organ damage which manifest at the molecular, cellular, tissue, and whole organism levels. **Table 1** and **Figure 1** highlight the critical molecular mechanisms involved while **Figure 2** shows how the systemic mechanisms interact. Each of the broad categories of IRI mechanisms considered below interact extensively to propagate the individual damage caused by the different mechanisms.

**Table 1:** Enumeration of the various molecular mechanisms at play during ischemia reperfusion injury, their various mechanisms of activation, how their activation propagates injury, and the end effect of the activation.

Molecular Mechanism	Mechanism of Activation	Result of Activation	End Effect
Calcium Overload	Hypoxia→Anaerobic Respiration→H+ imbalance→compensatory intracellular hyper-Ca2+	Activation of mPTP Inflammasome activation	Cell structure degradation Inflammatory transcription factor promotion
Reactive Oxygen Species (ROS)	Oxygen influx at reperfusion overwhelms ROS scavenging mechanisms, mPTP opening→ROS release from mitochondria	Alter cell-cell signaling Disruption of homeostatic intracellular protein activation Direct protein and DNA damage	Apoptosis Necrosis Amplify injury and inflammation Leukocyte activation
Cell Adhesion Molecules	Increased expression following reperfusion	Promote interaction between activated leukocytes and endothelium	Innate inflammatory reaction
PAMPs/DAMPs	Released from damaged cells→Recognized by pathogen recognition receptors	Proinflammatory molecule expression (ex: IL1β and IL18) Proapoptotic molecule expression in recruited leukocytes	Inflammation Apoptosis
Complement	Classical, alternate, and lectin mediated pathway activation	Membrane attack complex-based cell disruption Byproducts (C3a, C5a)→leukocyte attraction and inflammasome creation	Inflammation propagation Cell death
Mitochondria dysfunction	Hypoxia→Anaerobic respiration→Lactate and Succinate buildup→electron transport chain reversal→mPTP opening→mitochondrial damage	mPTP opening→ROS release Mitochondrial fission	Apoptosis Endothelial dysfunction
Endothelial dysfunction	ROS, Calcium overload, Mitochondrial damage→Endothelial cell damage→Tight junction phosphorylation, adhesion molecule upregulation, immune cell activation, vasoconstriction	Recruitment of leukocytes Activation of leukocytes Decreased barrier function Local thrombosis	Inflammation Further ischemia Cell and organ dysfunction and edema

Allo- and xenotransplant IRI presumably share some of these mechanisms. However, the relatively short cold ischemia intervals typical of experimental xenotransplant research studies would almost never be associated with graft dysfunction in clinical practice or in preclinical allograft models (25, 26). Recent evidence suggests that IRI mechanisms may be amplified by xeno-rejection mechanisms, including recipient innate anti-pig antibodies, interspecies complement dysregulation, coagulation cascade incompatibilities, and perhaps by other features of the profound innate xeno-immune response (25, 27).

## Innate Immune Cellular and Molecular Mechanisms of IRI

### Intracellular Calcium Overload/Calcium Paradox

Ischemia induces anaerobic respiration, which results in intracellular lactate and acid accumulation which decreases intracellular pH. Membrane Na^+^/H^+^ exchange pump activity increases to mitigate this (28–32). Intracellular hypernatremia then leads to an increase in cell membrane Na^+^/Ca^++^ exchanger activity, increasing the intracellular calcium levels (28–33). Ischemia also causes loss of adenosine triphosphate (ATP), which leads to dysfunctional intracellular organelle and cell membrane ATP-dependent calcium pumps, further exacerbating calcium overload (29, 31, 33). Upon reperfusion, the extracellular acid is washed out, increasing the proton gradient across the cell membrane and resulting in accumulation of cytosolic calcium (13, 28). This sequence of events was first observed by Zimmerman et al. in rat hearts; he termed this the ‘calcium paradox’ because although calcium was thought to be necessary for cardiac myocyte function, its reintroduction was detrimental during the reperfusion phase (34). This cytosolic hypercalcemia with immediate return of normal intracellular pH activates the mitochondrial permeability transition pore (mPTP), calpains, and calcium-dependent kinases which lead to lethal cell structure degradation, uric acid formation with inflammasome activation, inflammatory cytokine formation, and inflammatory transcription factor promotion (31, 35, 36). The fluctuations in pH and the ‘calcium paradox’ are particularly important in kidney, liver, and heart IRI given the importance of pH and calcium balance in normal cell and organ function (24, 31, 37–39).

### Reactive Oxygen Species

Reactive oxygen species (ROS) such as superoxide, hydroxyl radicals, peroxides, and singlet oxygen play a significant role in IRI (23, 24, 40, 41). Low levels of ROS are a physiologic mechanism of cell-to-cell signaling (42). The double bond in molecular oxygen provides a critical energy source that is normally harnessed by the mitochondrial oxidation-reduction system where the byproducts of converting oxygen and carbon-bound hydrogen molecular species to carbon dioxide and water maintains cellular oxygenation and reduction homeostasis (42, 43). Reperfusion causes a massive oxygen influx that generates significant quantities of free radicals (28). ROS generation in excess of physiologic ROS scavenging mechanisms coupled with acidosis leads to mitochondrial dysfunction and eventual rupture. ROS-mediated mitochondrial injury combined with mPTP opening driven by cytosolic hypercalcemia leads to an additional burst of ROS release from mitochondria (31, 44, 45). This bolus of ROS without functional protective scavenging molecules exceeds the capacity of ROS-degrading enzymes, and disrupts cellular function through three main pathways: 1) alteration of cell-cell signaling, 2) disruption of the oxygenation/reduction balance causing alteration of intracellular proteins, and 3) direct damage to cell proteins and molecules such as DNA (31, 42, 43). These three general mechanisms of damage can lead to cell death *via* apoptosis or necrosis (46).

ROS and oxidative stress are particularly important in allotransplantation (23, 24, 37, 47, 48). In lung allografts, ROS formed consequent to adenosine A2A receptor activation trigger activation of invariant NKT (iNKT) cells, leading to epithelial and endothelial damage (49–53). ROS are one of the initial mediators of damage to hepatocytes and liver sinusoidal cells during liver allograft reperfusion (31). The heart is also particularly sensitive to ischemia and subsequent oxidative stress during reperfusion secondary to its high oxygen demand and rapid exhaustion of intracellular ATP stores with brief periods of normothermic ischemia (38, 39, 48, 54, 55).

In the context of xenotransplantation, ROS are also presumed to play a role in xenotransplantation ischemia reperfusion injury (XIRI). *In vitro* data from rat livers perfused with human blood showed that hyperacute rejection requires reactive oxygen species, with additional primary or secondary contributions from leukocytes and complement activation (56). Additionally, hydrogen peroxide and extracellular calcium have been shown to strongly induce cell adhesion molecule expression on porcine islet xenografts, presumably amplifying injury, inflammation, and islet xenograft attrition (57).

### Cell Adhesion Molecules

Reperfusion following ischemia leads to increased expression of cell adhesion molecules including selectins and integrins on the endothelial surface of the injured tissue (58, 59). Upon reperfusion, endothelial cells and leukocytes activated by ROS and other pathways interact through these adhesion molecules *via* ligand proteins such as P-selectin glycoprotein ligand and sialyl LewisX (60, 61). This interaction is the basis of the innate inflammatory reaction associated with IRI in allotransplantation. Blocking selectin and integrin interactions in pre-clinical allotransplant models reduces both tissue damage and elaboration of pro-inflammatory chemokines into the circulation (59, 62).

### Pathogen and Damage Associated Molecular Proteins

Cell damage triggered by the ‘calcium paradox’ and/or ROS elaboration triggers the release of multiple pro-inflammatory mediators and cytokines including chemotactic cytokines (chemokines), as well as activation of complement cascade proteins. The most potent proinflammatory mediators are broadly classified as damage-associated molecular proteins (DAMPs), such as various adenine phosphate nucleotides (ATP, ADP, and AMP). Heat shock proteins (HSPs) are also released. HSPs are produced during oxidative stress to ensure proper folding of other proteins and act as DAMPs (63, 64). DAMPs differ from bacterial- and virally derived pathogen associated molecular proteins (PAMPs) because they are endogenous molecules that are normally carefully prevented from release into the circulation. Like PAMPs, DAMPs are recognized by pattern recognition receptors (PRRs) such as Toll Like Receptors (TLRs). Among PRRs, DAMPs are particularly efficient in activating NLRp3 (65–68). TLRs have been generally associated with both immediate inflammatory responses (such as those implicated in IRI), and with later kidney fibrosis, thought consequent to amplifying inflammation with secondary recruitment of T and B cells (37). TLR2 and TLR4 are of particular interest as PRRs mediating DAMP-driven injury because genetic deletion of one or both decreases severity of kidney IRI in a mouse model (69). When PRRs recognize PAMPs and DAMPs, there is additional upregulation of a variety of transcription factors, most importantly NF-κB, which increase the expression of proinflammatory and pro-apoptotic molecules and proteins within the recruited immune cells (31, 66, 70–73). NLRp3 activation leads to assembly of a caspase 1 activating platform called the inflammasome with subsequent release of active forms of IL1β and IL18 to promote the sterile inflammatory response characteristic of IRI (66, 70, 71). For example, formation of the inflammasome has been demonstrated in association with myocardial damage during heart IRI (66, 74–76). The NF-κB pathway and the inflammasome are critical mediators common to the multiple upstream pro-inflammatory pathways within the systemic sterile inflammatory response that is characteristic of IRI (77).

### Complement

Each of the three complement pathways (classical, driven by antibody binding and Fc-mediated complement activation; alternative, triggered by spontaneous unknown mechanisms; and lectin-mediated) can participate in IRI (31, 78, 79). Each pathway leads to formation of the C5b-9 ‘membrane attack complex’, the final common complement activation pathway which physically disrupts the cell membrane, overwhelming the ability of ion channels to maintain membrane polarization and osmolar gradients, resulting in cell death (31). Upstream byproducts of complement activation such as C5a and C3a are chemoattractants for neutrophils and macrophages which can lead to tissue inflammation (68, 72). Additionally, C3a and C5a can bind a variety of G protein coupled receptors as well as TLR2, which trigger NF-κB activation and the formation of the inflammasome (80, 81). Complement activation and deposition during reperfusion plays a role in IRI after acute MI, as well as in acute peripheral artery occlusion and in allografts, particularly kidney and liver (82–84). Accordingly, inhibiting complement activation has shown therapeutic benefit in animal models of each of these conditions (82–88). Clinical translation, however, has been slow and remains in preclinical and early-stage clinical trials (89–91).

In wild type pig-to-non-human primate (NHP) xenotransplantation, complement activation by the direct pathway (antibody-driven complement activation) is a central mechanism contributing to the phenomenon of hyperacute rejection (HAR) (92). Prolific complement activation associated with HAR was mitigated by two key genetic engineering accomplishments. First, human complement pathway regulatory protein (hCPRP)-transgenic pigs were created to enhance pig endothelial cell (EC) protection from human complement-mediated injury. Later, the porcine Gal-α1,3Galactosyl transferase gene was knocked out (GalTKO) to eliminate the predominant carbohydrate antigen recognized by human-anti-pig antibodies (93–95). Addition of one or more hCPRP’s to GalTKO further decreased the incidence of initial graft dysfunction by constraining amplification of the antibody-mediated classical complement cascade, inhibiting formation of the membrane attack complex, and better preventing endothelial injury relative to either hCPRP or GalTKO alone (96, 97).

### Mitochondrial Dysfunction

Mitochondrial dysfunction plays an important role in IRI and subsequent cell death. Mitochondrial dysfunction starts during ischemia, with accumulation of lactate and succinate from anaerobic respiration damaging molecules in the electron transport chain (98). Upon reperfusion, the accumulated succinate leads to electron transport chain reversal and ROS formation (98). This reduces the mitochondrial membrane potential leading to mitochondrial Ca^2+^ bursts, and mPTP opening, as discussed above (98). mPTP opening causes structural damage in the form of mitochondrial swelling and is a key step in irreversible cell injury after IRI, which has been especially well-studied in heart IRI (13, 44, 45, 55, 98, 99). While ischemia and the associated lactate accumulation decreases intracellular pH and prevents mPTP opening, reperfusion rapidly returns the intracellular pH to normal, contributing to mPTP opening, particularly when combined with increased intracellular ROS and calcium (13, 100–103). The open mPTP allows H^+^ to enter the inner mitochondrial matrix, further uncoupling the electron transport chain and decreasing ATP production (104–106). Decreased ATP and increased mitochondrial ROS secondary to mPTP opening can lead to mitochondrial fission, a canonical step in initiating apoptosis (13, 31, 107). Mitochondrial fission also results in dysfunction of other cell organelles, particularly in endothelial cells, leading to endothelial dysfunction, seen in both IRI and xenograft injury (108).

### Endothelial Dysfunction

Healthy endothelial cells are essential to maintain organ homeostasis and represent the first barrier between donor tissue parenchyma and recipient immune cells. Normal endothelium facilitates nutritive blood flow and waste clearance by preventing non-physiologic platelet adhesion and regulating local coagulation cascade activation (31). IRI disrupts multiple normal EC functions. Inflammatory cytokines, chemokines, proteases, histamine, and other proinflammatory mediators cause EC tight junction phosphorylation and internalization as well as calcium dependent myosin light chain phosphorylation, which leads to cytoskeletal contraction and loss of both paracellular and transcellular vascular barrier function (31, 109–113). Dysregulation of sphingosine-1-phospate, an endothelial tight cell junction protein, has been noted to play a significant role in IRI, most thoroughly studied in lung endothelial IRI (114).

Dying or dead cells release the ROS created after reperfusion. ROS enhance adhesion between leukocytes and endothelial cells by means of upregulation or activation of selectins and integrins, including ICAM-1, P-selectin, JAM-A, JAM-C, and PECAM-1 (31, 115–121). Similarly, multiple matrix metalloproteinases (MMPs) such as MMP9, are inducible gelatinases that disrupt liver sinusoidal endothelial cells and promote leukocyte adhesion *via* changes to PECAM-1 (122). Macrophages and mast cells are then activated, releasing chemoattractants such as TNFα, other proinflammatory cytokines, platelet activating factor (PAF), and the lipid mediator LTB_4_ (31). These mediators amplify recruitment, adhesion, and activation of neutrophils, which release chemokines and cytokines that further propagate a pro-inflammatory response and recruit other immune cells to the site of injury (112). Liver sinusoidal endothelial cells are normally protective against inflammation by their expression of transcription factors KLF2 and NRF2, but IRI damages these cells, which results in degradation of the anti-inflammatory transcription factors (41, 123, 124). In addition to elaboration of pro-inflammatory cytokines, expression of angiogenic growth factors such as VEGF is increased during IRI, and contributes to increased endothelial permeability during IRI of the lung, heart, and liver but not kidney (125–134).

ECs, damaged by pro-inflammatory cytokines and ROS, decrease production of endothelial nitric oxide (NO), a powerful vasodilator, which therefore results in vasoconstriction (135, 136). The decrease in EC NO release, coupled with upregulation of cell adhesion molecules, promotes platelet activation and adhesion, creating a prothrombotic local environment (137, 138). This leads to microthrombi formation, production of transcription factors for inflammatory mediators, and upregulation of costimulatory proteins such as CD28-B7 and ICOS-ICOSL that further propagate inflammation and cell/tissue death. They also contribute to amplification of adaptive immune responses to ‘non-self’ antigens, whether allo or xeno (117, 139, 140). Hypoxia inducible factor 1 (HIF-1) normally protects endothelial barrier function and vasodilatory function through modulation of VEGF and nitric oxide, but its dysregulation during ischemia is a significant factor in IRI (132, 141, 142).

In addition to endothelial injury, in all organs, but particularly in the lungs, epithelial injury increases levels of the receptor for advanced glycation end products (RAGE). HMGB1 is a traditionally intracellular protein that regulates a variety of nuclear functions, however upon ischemia induced necrosis, it becomes extracellular and acts as a DAMP. HMGB1 by itself or bound to inflammatory cytokines binds to membrane RAGE and becomes endocytosed. HMGB1 and/or its accompanying molecule then signal TLR4 which leads to activating the NF-κB pathway (143–145).

As in allotransplants performed in the setting of preformed anti-donor antibody (ABO mismatch, presensitized), in xenotransplantation, endothelial damage is initiated by pre-formed antibodies, in this case directed against pig carbohydrate and other antigens, that bind to the xenograft endothelium upon reperfusion, and are a critical initiator of XIRI. The pre-formed anti-pig antibodies activate complement and trigger Fc-mediated inflammatory cell adhesion and activation, amongst other inflammatory responses that damage the endothelium (146). Endothelial activation and consequent loss of thromboregulatory, anti-inflammatory, and anticoagulant functions propagates an already robust inflammatory response secondary to prolonged systemic inflammation in xenograft recipients (SIXR), which has been implicated as the culprit explaining the apparent requirement for an expanded spectrum of immunosuppressive treatments in successful xenotransplantation experiments (27).

Of particular note, the inflammatory mediators associated with SIXR are similar to those associated with XIRI (27). The importance of these inflammatory mediators in XIRI is highlighted by the especially robust response of immune cells to cross-species inflammatory mediators (147). French et al. explored this concept in IL8 and neutrophil interactions in an *ex vivo* pig-to-human lung xenotransplant model and *in vivo* pig-to-baboon lung xenotransplant model (147). These studies demonstrated that compared to allo- and autotransplantation, the xenotransplant model had significant elevation of baboon IL8 as early as four hours post-reperfusion (147). Additionally, human neutrophil adhesion was more robust when the neutrophil was activated by pig IL8 compared to human IL8 (147). Human TNFα activation of pig aortic endothelial cells also resulted in greater neutrophil adhesion compared to the neutrophil adhesion seen with human TNFα activation of human aortic endothelial cells (147).

Physiologically inappropriate formation or propagation of clot is also a feature shared between IRI and xenograft rejection due to hypoxia and inflammatory cytokine-induced upregulation of tissue factor production from endothelial cells (148). Tissue factor is a procoagulant enzyme that activates the extrinsic coagulation cascade. Intrinsic factor activation has also been implicated in the procoagulable state associated with IRI, especially in xenotransplantation (149). The procoagulable state associated with antibody binding and complement activation is amplified by a disturbance of the homeostatic balance between endothelial plasminogen activator inhibitor-1 to tissue plasminogen activator (150). As a consequence, transgenic expression of human tissue factor pathway inhibitor (hTFPI) in pigs is being studied to limit this coagulation dysregulation (151).

Dysregulated coagulation across species is known to contribute to xenograft injury, and has been addressed by expressing human coagulation pathway regulatory proteins (e.g., thrombomodulin, hTBM; endothelial protein C receptor, hEPCR; tissue factor pathway inhibitor, hTFPI) on pig endothelium (152). Xenografts from pigs with GTKO and complement regulatory transgenes, with or without additional coagulation pathway regulatory proteins, are the longest survivors to date in pig-to-NHP xenotransplantation, suggesting that the additional expression of one or more human coagulation pathway regulatory molecules may protect xenografts from IRI (25, 153, 154). Ultimately, genetic modification of the pig endothelium to prevent XIRI may prove to be critical in enabling successful clinical xenotransplantation (96, 155–157).

### Cell Death Mechanisms

Cell death occurs by four basic means: necrosis, apoptosis, autophagy, and necroptosis (31).

#### Necrosis

Necrosis is an uncontrolled or uncoordinated form of cell death generally triggered by extrinsic toxic exposures that damages the cell and disrupts biochemical functions essential to cell survival.

#### Apoptosis

Apoptosis is considered “programmed” cell death, and is often normal, coordinated, and beneficial, but may also occur in response to harmful stimuli. It is a controlled process of removing and recycling cells without causing inflammation or harming the organism. It occurs as the membrane blebs, shrinking the cell, while the nucleus collapses, chromatin condenses, and DNA fragments, until the remains are engulfed by macrophages. This process is mediated by a cascade of caspase proteases that result in cleavage of intracellular substrates. There are intrinsic and extrinsic pathways to initiate the caspase cascade. Importantly, apoptosis can be initiated by hypoxia *via* the intrinsic (mitochondrial) pathway, a key component of ischemic injury, as well as by ROS from reperfusion (158).

#### Autophagy

Autophagy, or “eating of self” is similar to apoptosis in that is also a noninflammatory process. As the cell begins to die, it degrades its own components *via* lysosomes (159). This is initiated by formation of a phagophore around intracellular contents to create an autophagosome, which fuses with the lysosome for degradation (159). This process is seen in both phases of IRI (160). ULK1 is a key component in autophagy, and is activated when the cell is depleted of nutrients and energy in ischemia (158).

#### Necroptosis

Necroptosis is an intermediate phenomenon, an amalgam of mechanisms that overlap between coordinated and uncoordinated cell death (31). It is essentially a “programmed” form of necrosis, triggered by RIPK3, seen in response to viral or intracellular bacterial infections and inflammatory diseases. This contrasts with apoptosis in that caspases are uninvolved and that the result is disorganized leakage of intracellular contents, including DAMPs, triggering innate and adaptive immune responses and causing inflammation (161). This is important in IRI, and RIPK3-deficient mice are protected from IRI (162).

#### Cell Death in Xenotransplantation

Cell death mechanisms in xenotransplantation are starting to be studied and apoptosis has been identified as one of the critical pathways (27, 163). Inhibition of the NF-κB pathway has been shown to lead to decreased porcine endothelial cell death/apoptosis in an *in vitro* model of xenotransplantation, whereas endothelial cell apoptosis has been shown to lead to xenograft rejection (164). These studies, however, reviewed the impact of SIXR leading to apoptosis, and were not specifically evaluating the role of cell death in XIRI (164).

## Systemic Mechanisms of IRI

### Innate Immune System

#### Neutrophils

Leukocytes are recruited by the local release of inflammatory chemokines and cytokines, by endothelial expression of selectins, integrins, and bound antibodies, by products of clot formation such as platelets and fibrin, and by exposed basement membrane secondary to endothelial cell retraction (165). Subsequently, neutrophils release additional inflammatory mediators including IL1, IL6, IL8, IL11, IFNγ, TNFα, LTB_4_, as well as proteases, and monocyte chemotactic factor 1 (23, 112). IL6, IL8, and TNFα have all been associated with xenograft injury and failure in multiple models (166–168). The selectin-dependent, complement-driven migration of neutrophils into the parenchyma, endothelial dysfunction, parenchymal edema, and numerous other processes have been implicated in the condition of “No-Reflow”. No-Reflow is a clinical finding of lack of perfusion to the microvasculature despite reperfusion at the tissue level following a prolonged ischemic event. It has been established to be associated with IRI in particular of the heart and limb (65, 169–173). Blocking cell adhesion molecules, particularly selectins and integrins, disrupting chemokine and cytokine release, as well as complement inhibition has each been shown to attenuate IRI and prevent or reduce the severity of “No-Reflow” (174–178).

#### Macrophages and Mast Cells

Tissue resident macrophages are activated by ROS, PAMPs, and DAMPs (179). Activated macrophages release TNFα, which leads to upregulation of NF-κB and other transcription factors throughout the reperfused organ or tissue (31, 180). The resident macrophages of the liver, Kupffer cells, are activated by DAMPs as well as by hepatocyte damage and release of the proinflammatory ligand HMGB1 (181, 182). Kupffer cell activation can further exacerbate hepatocyte injury by promoting neutrophil recruitment and upregulating NF-κB (31, 182–185). Similarly, resident lung macrophages are activated during ischemia reperfusion and enhance epithelial and endothelial dysfunction and inflammation by the TLR pathway (77, 186–189). ROS, TNFα, IL17, and C-X-C motifs are released from the injured epithelium, activated macrophages, and iNKT cells (51, 143, 186, 190–192). These are powerful pro-inflammatory cytokines and chemokines that recruit leukocytes (143, 186, 190–192). ROS, complement, LTB_4_, and other molecular mediators of IRI activate mast cells as well (193). Mast cells are subendothelial and reside in nearly all tissues. Mast cell activation leads to release of various monoamines, proteases, TNFα, and other inflammatory mediators that result in edema, inflammation, and local hemorrhage (193).

Mast cells and resident tissue macrophages also play a significant role in xenograft dysfunction, and may aggravate XIRI (155). Burdorf et al. demonstrated in an *ex vivo* lung perfusion xenograft model that blocking thromboxane and histamine receptors, mediators released by peri-endothelial mast cells, led to a blunting of initial rise in pulmonary vascular resistance, decreased pulmonary edema, and delayed loss of vascular barrier function after reperfusion (155).

#### Platelets

During IRI, platelet activation occurs through a multitude of receptors including platelet collagen receptor, adenosine diphosphate receptor, glycoprotein-IIb/IIIa (GPIIb/IIIa), P-selectin, and G-proteins. Additionally, complement byproduct-mediated, histamine-mediated, and thromboxane-mediated pathways exist which lead to platelet activation (194–197). Activated platelets release ROS, serotonin, and platelet activating factors, as well as interact with leukocytes and endothelial cells, propagating sterile inflammation, coagulation, and IRI (194).

In addition to these processes, Fc receptor mediated platelet activation and complement mediated platelet activation are particularly critical in xenotransplantation (198). The interaction between the complement system, coagulation-fibrinolytic system, and platelets leads to thromboinflammation. In the long run this leads to consumptive coagulopathy and is a major barrier in xenotransplantation (198). These processes have been traditionally associated with SIXR however each of the component pieces leading to thromboinflammation are also activated during XIRI (198).

### Adaptive Immune System

The adaptive immune system has been found to play an important role in IRI (37, 199–203). Dendritic cells, which are stimulated by ROS and DAMPs, are pivotal to triggering and amplifying activation of adaptive immunity (37). However, it is clear that the adaptive immune response not only propagates injurious mechanisms in IRI but can also down-regulate IRI (37). CD4+ and CD8+ T cells secrete inflammatory cytokines and chemokines such as IL1, TNFα, and IL17 to recruit other cells (37). CD8+ cells amplify IRI independent of the T-cell receptor and antigen specific T-cell functions (204). IgM and IgG produced by B-cells also contribute to IRI by ligating complement, neutrophils, macrophages, and platelets to xenoantigens in the graft and after release of cells and cell fragments into the circulation (37). The role of T-regulatory cells is an active avenue of research and they are thought to provide anti-inflammatory regulatory function in reducing IRI (37, 205, 206). NK and NKT cells are also thought to play a role in IRI, specifically in xenotransplantation where they have been shown to adhere to and damage the endothelium because of absence of self-recognition molecules (CD47, HLA-E), similar to a mechanism found in IRI-damaged or neoplastic cells (207).

### Brain Death

Brain dead donors constitute a significant portion of the allograft donor pool, including more than 50% of kidneys, 85% of livers and lungs, and almost all hearts (208). Brain death induces a sterile inflammatory response that has been well-characterized, and activates many of the same pathways that mediate IRI (209). These pathways include pro-inflammatory cytokine release, endothelial injury, coagulation pathway dysregulation, and enhanced leukocyte adhesion and migration in the lung and other tissues (210–213). Additionally, neuropeptides, such as neuropeptide Y, calcitonin gene related peptide, and substance P, released during brain death, propagate sterile inflammation within the donor (209, 214). Brain death also causes dysregulation of the hypothalamic-pituitary-adrenal axis resulting in decreased adrenocorticotropic hormone and cortisol (215–217). There is also a rapid depletion of antidiuretic hormone resulting in diabetes insipidus, and thyroid stimulating hormone resulting in central hypothyroidism and subsequent decrease in serum T3 and T4 (215–217). These changes have significant electrolyte and hemodynamic consequences throughout the brain death period requiring special attention and treatment so as to preserve the donor organs (215–217). The injurious consequences of each of these “sterile inflammation” pathways differs from organ to organ, as exemplified by varying behavior of different organs from individual donors (209). Sterile inflammatory mediators elaborated in association with brain death “prime” transplanted organs for a ‘second hit’, such as IRI (218). For renal allografts, where there is a robust reference group of case-matched controls, allografts from brain dead donors demonstrate a higher incidence of primary graft dysfunction and reduced graft survival relative to living donors when all other variables are taken into account such as graft ischemic time, HLA mismatch, and immunosuppression regimen (219, 220). The brain death priming process is blunted in organs procured from donors after cardiac death (DCD). The effects and mechanisms of IRI from DCD organ procurement, and the competing risk of extended warm ischemic time between cardiac death and organ flush *in situ*, still need to be elucidated (40, 221–226). With regards to known mechanisms contributing to IRI, xenotransplantation has the advantage of avoiding the “brain death priming” phenomenon seen in allotransplantation.

## Organ-Specific Ischemia Reperfusion Injury Minimization (IRIM)

Specific organ allografts have unique properties that make them more or less susceptible to different mechanisms of IRI (23, 24, 40, 41, 66, 165). In allotransplantation, there is now significant research on organ-specific mechanisms contributing to IRI, and IRIM strategies (23, 24, 40, 41, 66, 165, 227). Current research in IRIM for heart, lungs, kidney, and liver allografts include reducing the effects of ROS, and directly or indirectly inhibiting inflammatory mediators, complement, immune cell platelet adhesion and activation, and maintaining, preserving, or restoring endothelial barrier and vasoregulatory functions (23, 41, 50, 52, 114, 190, 227–265). Promising findings in reduction of lung IRI have included adenosine A2A receptor activation reducing microvascular permeability and lung injury, early growth response 1 (Egr1) deletion reducing neutrophil infiltration, C3a receptor antagonist decreasing cell injury and inflammation, carbon monoxide in cold flush reducing inflammatory mediators and cellular infiltrate, and nitric oxide to reduce pulmonary arterial pressures, inflammation, and apoptosis (50, 52, 190, 266–269).

Certain IRIM targets and treatments are common between multiple organs. Mesenchymal stem cell (MSC) treatment at the time of procurement, or during *ex vivo* perfusion before implantation, is a novel area of research in IRIM. MSCs are collected from bone marrow, amniotic tissue, or the umbilical cord, as their extracellular vesicles include apoptotic bodies, which were hypothesized to interact with other cells to quiet the inflammation seen in IRI (227, 228, 270). These have been administered by culturing the cells (with or without separation of extracellular vesicles) and adding to a perfusate such as Steen solution in *ex vivo* perfusion, as an intravenous infusion to the recipient at the time of reperfusion, or bronchoscopically (227, 239–241, 244, 246, 270–273).

MSC treatment has been found to be broadly protective of IRI in the heart, lungs, and kidney in preclinical models (23, 227, 239–241, 246, 270–274). It has been proposed that MSCs may have their protective effect by three general actions: paracrine secretion of soluble factors, increasing cell to cell interactions *via* microtubules, and secretion of vesicles containing proteins and nucleic acids (275). There is currently one phase 1 clinical trial using MSCs in kidney transplantation; early results have shown safety of MSC infusion in these patients (276). We expect the number of these studies to increase as has been done for in other disease processes that have trialed MSC therapy (277–279). Similarly, use of inhaled pharmacologic agents, specific inhaled anesthetic, or either inhaled or perfusate dissolved gasses such as nitric oxide, hydrogen sulfide, and carbon monoxide have been used in preclinical models to mitigate IRI of the lungs, liver, and kidneys (266–269, 280–284).

### Lung

In lung transplantation, primary graft dysfunction (PGD) is closely associated with duration of cold and warm ischemia, and is usually attributed to IRI (285, 286). *Ex vivo* lung perfusion (EVLP) for normothermic organ preservation and rehabilitation is currently in use clinically and has been found to potentially reduce IRI (287–289). EVLP was introduced to the clinic in 2011 for high-risk lungs (290). Preclinical studies in a porcine model have shown that EVLP may reduce IRI by decreasing the insult on the endothelium or by reducing the inflammatory cytokine load (291–293). Controlling reperfusion by means of reversing oxygen debt in the absence of mechanical stress/strain and in the absence of platelets and neutrophils are also beneficial attributes of EVLP (291, 292). When comparing tissue cytokine levels of EVLP lungs versus cold static preserved lungs from biopsies obtained two hours post implantation, EVLP lung tissue had significantly lower concentrations of IL1β, IL18, and IFNγ. This indicates that ischemia minimization is associated with reduced elaboration of pro-inflammatory cytokines. In this study, the potential IRI reduction may have been secondary to decreasing the injury mechanisms associated with cold storage and subsequent reperfusion (291). EVLP has the potential to significantly reduce IRI by improving organ preservation by reducing or preventing oxygen debt and provides a platform for delivering preventive and/or adjunctive reparative treatments based on defined IRI mechanisms.

### Kidney

In kidney transplantation, PGD encompasses both the relatively common delayed graft function (DGF), with recovery of glomerular filtration rate in days or weeks after transplant, as well as primary non-function, which does not recover. Renal allograft PGD is closely associated with duration of cold and warm ischemia, and is usually attributed to IRI (24). Inhibition of apoptosis, TLR signaling, and complement activation/injury are the IRIM strategies supported by strong preclinical and clinical evidence base, and are closest to clinical practice. QPI-1002 is an siRNA that temporarily inhibits p53 (37, 274). p53 inhibition in renal tubular cells has been shown to reduce apoptosis (37). QPI1002 was successful in reducing incidence and severity of DGF in phase I and II trials, and is currently in a phase III clinical trial to reduce DGF in kidney transplant patients (NCT0261096) (37, 294). An anti-TLR2 monoclonal antibody (Tomaralimab, Opsona Therapeutics Ltd, Dublin, Ireland) is currently in phase II clinical trials for DGF reduction (37, 262). Recombinant C1 inhibitors (C1-INH) are being evaluated in multiple clinical trials, with interim reports suggesting equivocal effects and other studies showing reduced severity of DGF (37). Complement Receptor 1 (CR1) is a protein found on the surface of many different cell types that downregulates C3 convertase (24). Mirococept (APT070, Inflazyme Pharmaceuticals, Richmond, BC, Canada) is a CR1 with a membrane anchor currently in phase III clinical trials for preventing IRI in kidneys (295). Orthosteric inhibition of leukocyte integrin CD11b/CD18 to diminish kidney IRI is an active area of research in a non-human primate model (296). Hypothermic (1-8°C) *ex vivo* perfusion is a long-established technology, and is a widely used method in many organ procurement organizations based on evidence of improved outcomes, including reduced DGF for deceased donor kidneys from high-risk donors or with expected prolonged cold ischemic times, intending to reduce IRI (253). More recently, normothermic blood based *ex vivo* perfusion has been shown to reduce the incidence of DGF in kidney allografts and this protocol is now in phase II clinical trials with primary outcomes being the incidence and nature of renal allograft DGF (ISRCTN15821205) (297, 298).

### Liver

Liver PGD (early allograft dysfunction [EAD] or primary nonfunction [PNF]) is generally ascribed to IRI (299). Machine perfusion (MP) has been studied most extensively in preclinical models to combat IRI, paving the way for clinical trials (22, 300–312). The three main techniques of MP that have become clinically applicable include hypothermic MP, hypothermic oxygenated MP, and normothermic MP (NMP). Each technique aims to mitigate IRI by reducing standard cold storage time and associated ischemic injury. In recent trials, the use of MP has been shown to reduce histologic bile duct injury and decrease post-operative hepatocellular enzyme release, both surrogates for IRI (22, 302, 304). Of interest, the first ischemia-free liver transplant was performed in China, where NMP was initiated in the donor and continued without interruption until graft revascularization in the recipient (313). This technique is hypothesized to have reduced IRI given minimal hepatocyte necrosis and apoptosis seen on post-reperfusion graft histology, low levels of inflammatory cytokine release by immunohistochemical staining and quantitative real-time polymerase chain reaction, and low post-reperfusion hepatocellular enzyme levels.

### Heart

Heart allograft primary graft dysfunction has been linked to IRI and manifests as global biventricular dysfunction – ranging from diastolic dysfunction in milder cases to progressively depressed systolic function in its more severe presentation (314). PGD after cardiac transplantation remains a significant clinical problem despite decades of research (315). Targeting inflammatory cytokines may be a new and promising avenue for IRIM. Tocilizumab (Genentech/Roche, San Francisco, CA, USA), an anti-IL6R monoclonal antibody, has proven efficacious in a clinical trial in reducing myocardial inflammation after acute myocardial infarction (316). It is currently being investigated in the heart transplant setting with study endpoints being donor specific antibodies, acute cellular rejection, antibody mediated rejection, hemodynamic compromise secondary to clinical rejection, death, or transplantation in the first year (NCT03644667). Antibodies against IL1R and TNFα reduce associated heart IRI in preclinical models but have not yet been evaluated in clinical trials (317, 318). The availability of clinically approved anti-IL1R and anti-TNFα antibodies (Anakinra, Swedish Orphan Biovitrum AB, Stockholm, Sweden and Etanercept, AMGEN, Thousand Oaks, CA, USA) make these avenues especially enticing. Heart IRIM *via* normothermic *ex vivo* perfusion has clinical approval and is currently available (319–322). On the horizon for cardiac allotransplantation is hypothermic *ex vivo* perfusion. Nilsson et al. compared hypothermic *ex vivo* perfusion to cold static storage in a recent phase II clinical trial and showed safety and efficacy of the circuit in humans (323). *Ex vivo* heart perfusion limits IRI by minimizing cold static storage time and thus minimizing organ ischemia.

Cardiac reanimation after DCD procurement is being used to expand the transplant donor pool (319–322). Optimizing the initial reperfusate of the heart contributed to the success of heart DCD (40). The DCD heart can be procured in two ways: direct procurement and preservation (DPP) and normothermic regional perfusion (NRP) (40). In DPP an emergent sternotomy is performed after declaration of death and the heart is perfused with cardioplegia solution during dissection. The heart is then explanted, connected, and reanimated with the normothermic *ex vivo* perfusion device. Throughout travel to the recipient’s facility the heart is perfused with normothermic oxygenated blood procured from the donor. The principles of the cardioplegia strategy for DPP and IRIM both aim to reoxygenate the myocardium, flush out ROS, replete ATP stores, and restore calcium homeostasis (40). In NRP, once death is declared, a sternotomy is performed, the head vessels are clamped, and the body is resuscitated *via* central or peripheral extracorporeal life support. The heart is then assessed *in situ* and if it is functionally normal, it is dissected, arrested, removed, and placed on the *ex vivo* perfusion device.

## Discussion

### IRI in Xenotransplantation

If shown to be dependably successful, organ xenotransplantation from pigs would provide an abundant and ethically acceptable alternative to human donors as a source of organs for transplantation that would be available on demand. Over the past 20 years, pigs have been genetically modified to address known xenograft injury mechanisms driven mainly by preformed antibody, complement activation, and coagulation pathway dysregulation. Long-term, life-supporting xenograft survival has recently been achieved using organs from several of these pig strains and transplanting them into non-human primates, the best available model for porcine-to-human xenotransplantation (4, 324). Three components of conventional allograft IRI that we believe are particularly critical to XIRI are complement activation, sterile inflammation, and endothelial activation (27, 146, 325, 326). However, heart xenografts of multiple different phenotypes and even pig hearts with three key xeno gene modifications (GalTKO.hCPRP.hTBM) were unable to dependably support life *in vivo* in the hands of multiple experienced investigators. Only with XIRI minimization were Langin et al. able to achieve consistent xenograft survival more than one day (25, 327, 328).

XIRI is a critical barrier to clinical xenotransplantation, but it affects xeno-organs to different degrees (25, 153, 329, 330). The xeno-heart seems to require robust XIRI minimization technique to avoid perioperative cardiac xenograft dysfunction (PCXD) (25, 153, 328). Additionally, Shah et al. showed that liver xenotransplant recipients suffer lethal coagulopathy, and Watanabe et al. showed that lung xenotransplantation is characterized by significant endothelial dysfunction and loss of vascular barrier function (alveolar hemorrhage) (329, 330). The mechanistic basis for the vulnerability of hearts and other organ xenografts to IRI may be related to xeno-specific mechanisms. For example, Khalpey et al. found that pig endothelial cells lose expression of ecto-5’ nucleotidase when exposed to human and non-human primate blood. This leads to loss of a key producer of extracellular cryoprotective, antithrombotic, and immunosuppressive adenosine which would put the porcine organ at increased risk of IRI (331). Porcine organ vulnerability may be due to this or a yet undiscovered peculiarity of pig physiology.

### Ischemia Reperfusion Injury Minimization in Xenotransplantation

Complement and coagulation pathway regulatory molecules, self-recognition receptors that inhibit cell-mediated injury, and constitutive expression of anti-inflammatory transgenes that have been incorporated into porcine donors to address xeno-specific injury mechanisms are likely to play an important role in minimizing IRI (**Table 2**) (156, 157, 332). Preventing detrimental complement activation, endothelial dysfunction, and reducing the cross species inflammatory response will be critical for future research into XIRI minimization. Langin et al. and Mohiuddin et al. have used pigs that express complement regulatory protein (hCD46) and pretreat their heart xenotransplant recipients with complement inhibitors. However, Langin reported that those interventions along with additional expression of human thrombomodulin, did not prevent initial xenograft dysfunction (IXD) (25, 333). Thus, while expression of these two human complement and coagulation pathway regulatory proteins would be expected to be protective for GalTKO hearts in baboons, ischemia minimization was necessary and sufficient, at least when anti-IL6R, anti-IL-1R, and anti-TNFα were also administered.

**Table 2:** Summary of the promising mechanisms of ischemia reperfusion injury minimization currently being used in *in vivo* and *in vitro* models.

	Current Model	Intervention	Results
Complement Regulation	Pig-to-NHP *in vivo* transplant	Porcine transgenesis of human complement regulatory proteins such as hCD46	Successful transgenic pig to NHP xenotransplantation (Heart, Kidney, Lung)
Coagulation Regulation	Pig-to-NHP *in vivo* transplant	Porcine transgenesis of coagulation regulatory proteins such as hTBM, hTFPI, hEPCR	Successful transgenic pig to NHP xenotransplantation (Heart, Kidney, Lung)
Anti-inflammatory Medications	Pig-to-NHP *in vivo* transplant	Anti-IL1, Anti-IL6R, Anti-TNFα	Successful transgenic pig to NHP xenotransplantation (Heart, Kidney, Lung)
*Ex vivo* Cold perfusion	Pig-to-NHP *in vivo* transplant	Cold continuous *ex-vivo* reperfusion followed by intermittent reperfusion during organ implant until cross clamp is removed	Successful transgenic pig to NHP xenotransplantation with long term heart xenograft survival
Self-recognition proteins	Pig-to-NHP *in vivo* transplant	Porcine transgenesis of self-recognition proteins such as hCD47, hHLA-E	Successful transgenic pig to NHP xenotransplantation (Heart, Kidney, Lung)
Mesenchymal Stromal Cells (MSCs)	Human-to-sheep myocardial infarction (MI) Human-to-Canine cardiopulmonary bypass (CPB)	Intracoronary injection of MSCs Intravenous injection of MSCs	MI model – improved myocardial perfusion in the treated group CPB model – decreased inflammatory cytokine levels
Heme-oxygenase-1	Pig-to-human *in vitro* oxidative stress model	Porcine transgenesis of HO-1 put under oxidative stress and by human TNF in *in vitro* conditions	Reduced reactivity to oxidative and human TNF.

The relative contribution of complement, coagulation, and inflammation to XIRI remains unknown. Xenograft injury and allograft IRI are both associated with pro-inflammatory cytokines (168, 316–318, 334–336). Whether the IL6, IL1, and TNFα pathways and complement and coagulation pathway dysregulation contribute significantly to IXD of heart xenografts has not been addressed and could be studied by omitting these interventions from the regimen, or by including these agents when using hearts without the hCD46 or hTBM genetic modifications.

As described by Langin et al. in the orthotopic pig-to-baboon heart xenotransplant model, organ preservation with machine perfusion is the most obvious and readily available approach to IRIM. In their initial experiments, standard cold static ischemia was associated with IXD in all but one of four consecutive xenografts. In contrast, when the hearts were perfused with cold (8°C) oxygenated cardiac STEEN solution (XVIVO, Gothenburg, Sweden), a hyperkalemic, hyperosmotic, blood-containing cardiac preservation solution developed by the Steen group, no IXD was reported for 9 consecutive subjects (25, 337). The perfusate was administered continuously to the donor heart prior to explant and either continuously or intermittently, at 20-minute intervals, throughout implant. Their perfusate contained hormones, inotropes, and anesthetic agents based on 20 years of work in the field. Delivery of an oxygenated perfusate in an arrested heart allowed for restoration of oxygen supply with minimal consumption; avoiding ischemia minimized ROS production. The continual circulation of an electrolyte and pH balanced solution likely minimized ATP depletion, mPTP opening, and calcium and other ion flux. The absence of platelets and leukocytes and their associated pro-inflammatory cytokine and lipid arachidonic acid-derived mediators may have also helped in preventing IRI. The pretreatment of the recipient with a cytokine antibody cocktail may have blocked the effects associated with release of these inflammatory mediators at initial xenograft reperfusion. These multiple different treatments, in combination, were sufficient to prevent graft dysfunction; whether they had direct or indirect effects to inhibit significant endothelial dysfunction, complement activation, and significant initial inflammation remains to be determined.

Normothermic *ex vivo* perfusion is used clinically in allotransplantation with good effect, particularly in liver and heart transplantation (338, 339). There are no current studies demonstrating the effects of normothermic *ex vivo* perfusion in xenotransplantation. Normothermic machine perfusion shortens cold ischemia while supporting the full organ metabolism allowing for assessment of injury and function. Hypothermic and subnormothermic machine perfusion have also been shown to enhance mitochondrial function and replenish cellular energy stores (338). Lastly, ischemia-free organ transplantation requires normothermic machine perfusion and completely abolishes ischemia. Given that there is no component of trauma or brain death with xenotransplantation, and ideally no question of organ viability before procurement, the role of *ex vivo*/machine perfusion and ischemia minimization in xenotransplantation require further investigation for all organs, and may be critical to safe, successful clinical translation.

Other strategies in XIRI minimization include the use of mesenchymal stem cells (MSCs). MSCs are hypothesized to mitigate IRI through their anti-inflammatory, pro-immunomodulatory, and tissue repair characteristics. Dayan et al. utilized a myocardial infarction model in sheep with treatment with human MSCs, showing improvement in myocardial perfusion at one month in the treated group without adverse events (270, 273). Qiang et al. utilized a canine model of cardiopulmonary bypass-induced IRI and treatment with human amniotic MSCs which led to mitigation of IRI as evidence by decreased levels of TNFα and IL8 and increased levels of IL10 relative to dogs not treated with MSCs (211).

Significant work has shown that activating the protective NRF2/heme oxygenase-1 (HO-1) pathway may reduce oxidative and inflammatory stress associated with IRI (340, 341). Bach et al. demonstrated the importance of HO-1 in a mouse-to-rat cardiac xenograft model, noting increased expression of HO-1 in xenografts with long-term survival (342, 343). *In vitro* work involving transgenic expression of human HO-1 into pigs has shown a decrease in reactivity of pig fibroblasts to oxidative stress as well as human TNFα, therefore this gene has been added in genetically engineered pigs (344). The separate effect of HO-1 on organ behavior in xenotransplantation has not yet been studied. Of note, delivery of an ROS scavenger may also reduce oxidative stress and attenuate XIRI, but further work is needed (345, 346).

Limiting cross-species coagulation dysregulation may help minimize XIRI. Anticoagulant genes should diminish coagulation pathway dysregulation and blunt inflammation. CD39 expression should improve ADP and AMP catabolism, reducing platelet and EC activation, and promote elaboration of anti-inflammatory adenosine by endogenous CD73, thus inhibiting both physiologically inappropriate clotting and inflammation (347). Of note, pigs are relatively deficient in CD73, perhaps accounting in part for the pig’s particular IRI susceptibility. Human endothelial protein C receptor (hEPCR) transgenesis along with hTBM and hTFPI are all likely to reduce dysregulated coagulation, and EPCR, interacting with PPAR-1, upregulates potentially critical anti-inflammatory intracellular mechanisms (348, 349). Including one or more of these genetic modifications in the pig construct may reduce the need for ischemia minimization strategies to reduce IRI and its consequences. Parenthetically, addressing porcine vWF-human GP1b abnormal interaction (blocking Fab; humanizing pvWF by genetic engineering) reduces, but does not prevent, platelet sequestration seen in heart, kidney, liver, and lung models (350).

Molecular mechanisms of IRI mentioned previously such as the calcium paradox, mitochondrial dysfunction, and mPTP have not been studied in the context of xenotransplantation but may be presumed to be similarly important. *Ex vivo* perfusion should minimize activation of these pathogenic mechanisms since the cellular electrochemical and energy imbalances associated with ischemia and reperfusion are reduced or prevented prior to transplantation.

Immune cell adhesion and activation also play a role in XIRI. Using genetic engineering to express self-recognition molecules on the porcine endothelium may help attenuate XIRI. Damaged or neoplastic cells lose expression of self-recognition receptors such as CD47 and HLA-E, resulting in activation of innate immune cell scavenging by neutrophils and monocyte/macrophage lineage cells (CD47), NK cells, and other myeloid cells (HLA-E) (351, 352). Similarly, pig cells that lack human CD47 and HLA-E are susceptible to phagocytosis or cytolysis by these recipient effector leukocyte populations. In response, CD47 has been expressed in pigs, and was associated with prolonged survival in a porcine-NHP lung xenotransplant model (329). Incorporation of hHLA-E into the porcine endothelium also suppresses NK cell and macrophage activation (351).

Selectin antagonism is effective in decreasing IRI in MI, peripheral arterial, and allograft IRI models. It seems logical that selectin blockade will similarly attenuate XIRI by decreasing the rolling and adhesion of neutrophils, although this has not yet been confirmed. Further work specifically for XIRI needs to be done in models where other xeno-driven cell adhesive interactions are addressed, allowing this specific question to be answered (146).

While complement inhibition and coagulation regulation have been mentioned with respect to genetic engineering, these mechanisms can be targeted by other methods. Soluble hTBM or direct thrombin inhibitors can be administered, and should work synergistically to membrane bound hTBM to decrease activation of the coagulation cascade (353). IL4 activates the PI3K/Akt pathway in endothelial cells and has been shown to be protective of complement-mediated endothelial cell damage in xenotransplantation *in vitro* models. IL4 transfection with or without *ex vivo* perfusion, may be another avenue for protection from complement mediated IRI injury (354).

In addition to the innate immune system, altering how the adaptive immune system responds to the xenograft may attenuate XIRI by use of conventional immunosuppressive agents or other approaches. Artificial antigen presenting cells have been used in oncology to stimulate anti-tumor specific T-cell responses, and could in principle be engineered to regulate how the recipient immune system responds to the pig antigen (355).

## Conclusion

IRI and its consequences for initial allograft organ function are well known to exert a critical negative influence on the initial and long-term outcomes of allotransplantation (23, 24, 41, 315). Minimizing IRI by *ex vivo* organ perfusion has allowed expansion of the donor pool in allotransplantation, and specifically enabled safer use of DCD organs. In addition, there is considerable evidence that IRI minimization by machine perfusion of ‘marginal’ kidneys, lungs, and livers is associated with improved allotransplant outcomes (288, 319, 356). To the extent that the major injury mechanisms are shared between xenograft injury and allograft IRI, including complement activation, endothelial dysfunction, and a profound inflammatory response, it is logical that genetic engineering modifications targeting these mechanisms should also attenuate XIRI (27, 146, 147, 155, 325, 326). Evidence to date indicates that XIRI is particularly critical in cardiac xenotransplantation. Efficacy of ischemia minimization in other xeno organs has not yet been evaluated (25, 153, 329, 330). By analogy with allograft experience, minimizing IRI either with *ex vivo* perfusion, or by targeting shared, overlapping injury mechanisms, may be critical for clinical translation. We suspect that using a combination of both approaches may offer critical advantages through redundancy to improve safety, maximize the odds of initial success with xenotransplantation in the short term, and the potential long-term benefits. With the advances of genetic modification, cytokine inhibition along with ischemia minimization *via ex vivo* organ perfusion has enabled a breakthrough in cardiac xenotransplantation. It remains to be seen which features are critical, and whether any drug treatments to the recipient can be eliminated using pigs with genes that constrain IRI mechanisms, like HO-1, HLA-E, hCD47. If not, continued progress in our understanding of XIRI mechanisms is likely to contribute to significant additional progress in pig-to-non-human primate XIRI minimization and xenotransplantation preclinical outcomes, providing hope for future clinical translation (25, 153, 329, 330).

## Author Contributions

PP: Performed majority of background research and writing of the paper. MC and TC: Participated in background research and writing of the paper. AC, FP, JFM, LB, AA, and JCM: Participated in writing of the paper. RP: Participated in concept development and writing of the paper. All authors contributed to the article and approved the submitted version.

## Funding

RP: NIH RO1 AI153612 (PI: RP) CRISPR-Modified Cardiac Xenograft Transplantation. FP: German Heart Foundation (Deutsche Herzstiftung e.V.).

## Conflict of Interest

The authors declare that the research was conducted in the absence of any commercial or financial relationships that could be construed as a potential conflict of interest.

## Publisher’s Note

All claims expressed in this article are solely those of the authors and do not necessarily represent those of their affiliated organizations, or those of the publisher, the editors and the reviewers. Any product that may be evaluated in this article, or claim that may be made by its manufacturer, is not guaranteed or endorsed by the publisher.

## Abbreviations

ATP, adenosine triphosphate; CR1, complement receptor 1; DAMPs, damage-associated molecular proteins; DGF, delayed graft function; DNA, deoxyribonucleic acid; DPP, direct procurement and preservation; DCD, donors after cardiac death; EAD, early allograft dysfunction; EC, endothelial cell; EVLP, *ex vivo* lung perfusion; GalTKO, gal-α1,3galactosyl transferase gene was knocked out; GPIIb/IIIa, glycoprotein-IIb/IIIa; HO-1, heme oxygenase-1; hCPRP, human complement pathway regulatory protein; hEPCR, human endothelial protein C receptor; hTFPI, human tissue factor pathway inhibitor; HAR, hyperacute rejection; HIF-1, hypoxia inducible factor 1; IXD, initial xenograft dysfunction; iNKT, invariant NKT; IRI, ischemia Reperfusion Injury; IRIM, ischemia reperfusion injury minimization; MP, machine perfusion; MSC, mesenchymal stem cell; MMPs, metalloproteinases; mPTP, mitochondrial permeability transition pore; MI, myocardial infarction; NO, nitric oxide; NHP, non-human primate; NMP, normothermic Machine perfusion; NRP, normothermic regional perfusion; PAMPs, pathogen associated molecular proteins; PRRs, pattern recognition receptors; PCXD, perioperative cardiac xenograft dysfunction; PAF, platelet activating factor; PGD, primary graft dysfunction; PNF, primary nonfunction; ROS, reactive oxygen species; RAGE, receptor for advanced glycation end products; SIXR, systemic inflammation in xenograft recipients; TLRs, toll like receptors; XIRI, xenotransplantation ischemia reperfusion injury.
